# Tumor microenvironment and immune evasion in head and neck squamous cell carcinoma

**DOI:** 10.1038/s41368-021-00131-7

**Published:** 2021-08-02

**Authors:** Areeg Elmusrati, Justin Wang, Cun-Yu Wang

**Affiliations:** 1 Laboratory of Molecular Signaling, Division of Oral Biology and Medicine, School of Dentistry, UCLA, Los Angeles, CA USA; 2 Jonsson Comprehensive Cancer Center, UCLA, Los Angeles, CA USA

**Keywords:** Cancer microenvironment, Oral cancer

## Abstract

Head and neck squamous cell carcinoma (HNSCC), an aggressive malignancy, is characterized by high morbidity and low survival rates with limited therapeutic options outside of regional surgery, conventional cytotoxic chemotherapy, and irradiation. Increasing studies have supported the synergistic role of the tumor microenvironment (TME) in cancer advancement. The immune system, in particular, plays a key role in surveillance against the initiation, development, and progression of HNSCC. The understanding of how neoplastic cells evolve and evade the immune system whether through self-immunogenicity manipulation, or expression of immunosuppressive mediators, provides the foundation for the development of advanced therapies. Furthermore, the crosstalk between cancer cells and the host immune system have a detrimental effect on the TME promoting angiogenesis, proliferation, and metastasis. This review provides a recent insight into the role of the key inflammatory cells infiltrating the TME, with a focus on reviewing immunological principles related to HNSCC, as cancer immunosurveillance and immune escape, including a brief overview of current immunotherapeutic strategies and ongoing clinical trials.

## Introduction

HNSCC is the sixth most common cancer, comprising 6% of all cancers worldwide, with an estimated incidence of approximately 650,000 cases arising each year and 350,000 cancer-related deaths annually.^1^ In the United States in particular, 3% of all malignancies are diagnosed as head and neck cancer, with over 53,000 new cases and a mortality rate of 10,800 deaths annually.^2^ HNSCC is an aggressive cancer characterized by increased mortality. However, the primary treatment modality for HNSCC is still limited to surgery with adjuvant standard radio or chemotherapy. Such cancers are difficult to treat and eradicate surgically due to the complexity of the maxillofacial anatomy, difficulties related to surgical access, and the need for preservation of functionality, which can also have associated morbidity due to esthetic and psychosocial impact on patients. Although head and neck cancers most often originate from squamous mucosa of the oral cavity, predominantly involving the tongue and floor of mouth as well as the upper aerodigestive tract (oropharynx, nasopharynx, and larynx), these cancers have heterogeneous characteristics with various entities. As a result, treatment approaches and outcomes differ significantly among the diverse subsites.

HNSCC has diverse risk factors, including excessive alcohol consumption, tobacco, diet, and genetics, which may influence risk independently or synergistically, and which vary among the different subsites of HNSCC occurrence. However, over the past two decades, although previously known to be a disease of middle aged and elderly, the incidence of HNSCC has been increasing in young patients and particularly in developed countries. This escalation has been partly attributed to tonsillar and oropharyngeal cancers related to an infection with high-risk strains of human papillomavirus (HPV), but the particular causes and mechanism of this rapid increase remains rather speculative.^3,4^

Worldwide, there has been a 26.6% increase in the incidence of HPV^+^ HNSCC reported cases; however, only up to half of HNSCC cases have been reported to be related to HPV infections within the United States.^5^ Both HPV^−^ and HPV^+^ HNSCC have distinct features. HPV^−^ HNSCC is characterized by mutations in tumor suppressor gene p53, a decrease in p16 expression, and an amplification of retinoblastoma (Rb). In contrast, HPV-driven tumors degrade p53, increase p16 expression, and inactivate Rb.^6^ Moreover, HPV^−^ cancers commonly present with a loss of chromosome 9p, which is responsible for downregulation of p16 (CDKN2A) expression, and also duplication of chromosome 7p, promoting epidermal growth factor receptor (EGFR) overexpression. On the other hand, HPV-driven cancers express amplifications in chromosome 3q, a loss of chromosomes 16p, 16q, 14q, 13q, and 11q, and mutations in the P13KCA gene.^7,8^ Expression of oncoproteins E6 and E7 encoded by HPV viral DNA leads to ubiquitination of p53 and loss of Rb/E2F complex regulation of the cell cycle.^6^ In addition, HNSCC HPV^−^ and HPV^+^ tumors can be distinguished clinically.^9^ Unlike HPV^−^ tumors, HPV-driven cancers predominantly occur in young patients that have no history of smoking or excessive alcohol intake, most commonly occur within the tonsillar beds in the oropharynx, and are more radiosensitive, which likely contributes to better patient outcomes and survival rates when compared to patients with HPV^−^ tumors.^9,10^

In 2018, the latest published edition of the American Joint Committee of Cancer and the International Union of Cancer recognized the need for a separate staging system for oropharyngeal cancer based on HPV status. This was mostly dependent on the fact that HPV^+^ oropharyngeal cancer had a more favorable prognosis and distinct clinical features, behaving as a completely different disease than HPV^−^ oropharyngeal cancer.^11^ Current research on HNSCC has highlighted the importance of genetic analyses in understanding the molecular pathogenesis of the disease. While classical mutations including RAS and PIK3CA occur in these cancers, they are relatively rare. Recent studies have focused on the disruption of various tumor suppressor pathways such as Rb/INK4/ARF, p53, and Notch, and evidence indicates that these may contribute to dysplastic cell initiation and tumor progression.^12–14^

**Fig. 1:**
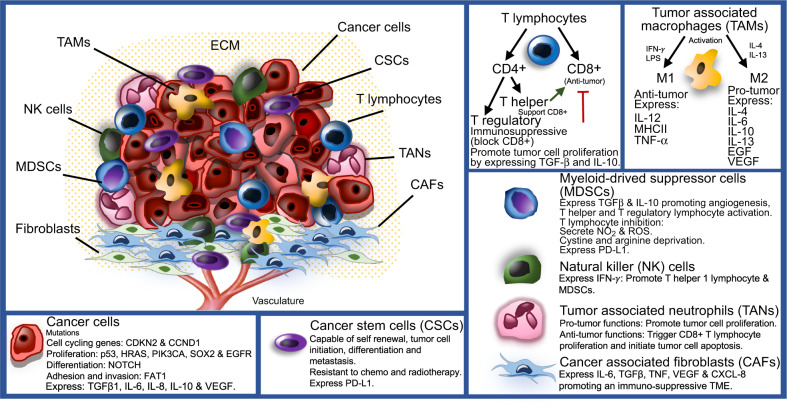
The TME of HNSCC. The TME is composed of cancer cells and heterogenous nonmalignant cells integrated in a complex extracellular matrix (ECM). The main cellular components of the TME are T lymphocytes, tumor-associated macrophages (TAMs), myeloid-derived suppressor cells (MDSCs), natural killer (NK) cells, tumor-associated neutrophils (TANs), and cancer-associated fibroblasts (CAFs). Immune cells play a key role in tumor cell growth and dissemination

It is well understood that all malignancies including HNSCC originate from the accumulation of genetic and epigenetic alterations and aberrations in cancer-related signaling pathways, leading to the development of cancer-related phenotypes that have been outlined previously.^15^ These hallmarks involve unlimited neoplastic cell replication, ability to avoid cellular apoptosis, resistance to anti-growth signals, self-sufficiency in growth signals, angiogenesis, invasion, and metastasis. Nonetheless, solid tumors are complex growths that comprise a network of neoplastic tumor cells and surrounding stroma in an extracellular matrix (ECM), referred to as the tumor microenvironment (TME) (Fig. 1). When investigating solid cancers, the TME and its interactions with the tumor are of great importance and must also be considered. Throughout the development and progression of cancer, the tumor and surrounding microenvironment are in constant communication and continuously evolve together, selecting for traits that favor tumor growth and invasion.

The immune landscape of the TME plays a key role in surveillance against development and advancement of HNSCC. Understanding the mechanism of immune system evasion in cancer is of utmost importance in the development of novel therapies and for the improvement of patient’s outcomes. In HNSCC, tumor cells can also utilize the immune cells in the TME to promote tumor cell proliferation, angiogenesis, and metastasis. These cells have the ability to escape the host immune system by hindering their own immunogenicity and expression of immunosuppressive signals. Recently being granted approval by the Food and Drug Administration (FDA), immune checkpoint inhibitors anti-programmed cell death protein 1 (PD-1)/PD-L1 are the first drugs to demonstrate prognostic benefits for the treatment of platinum-refractory recurrent or metastatic HNSCC.^16^ Investigation of other immune checkpoint receptors as cytotoxic T-lymphocyte-associated protein 4 (CTLA-4) solely or in conjunction with other immunotherapies or conventional cancer therapeutics has been gaining more attention. The objective of this review is to discuss recent insights into the tumor–stromal crosstalk in the pathogenesis of HNSCC, focusing on the roles of the immune system and inflammatory signal pathways within the TME in the regulation of tumor initiation, development, progression, and metastasis.

## Molecular pathogenesis and genetics of HNSCC

Cancer originates through the accumulation of genetic and epigenetic signaling pathway alterations in genes, causing the acquisition of different cancer phenotypes.^15^ One of the major advances in understanding the pathogenesis of HNSCC was reported by two independent groups, following a whole exome sequencing analysis of HNSCC specimens.^14,17^ Combining these findings with previous functional and genomic analyses, highlighted a distinct number of oncogenes targeted by mutations leading to the activation of tumor suppressor pathways, comprising p53, Rb/INK4/ARF, and Notch in HNSCC tumor pathogenesis. In general, these genes play key roles in cellular proliferation, differentiation, survival and metastasis.

In 2015, The Cancer Genome Atlas Network (TCGA) conducted a massive genetic study where they examined 279 primary HNSCC tumors. The study included the common sites of HNSCC (62% oral cavity, 26% larynx, and 12% oropharynx), 73% were male smokers, while 13% tested positive for HPV as defined by RNA expression of viral genes, primarily E6 and E7. This research also profiled protein expression, copy-number alterations, and epigenetic changes.^8^ In agreement with previous studies, TCGA gene mutations were separated into four groups comprising cell-cycle regulating genes (*CDKN2A* and *CCND1*), genes involved in regulating cell proliferation and survival (*TP53*, *HRAS*, *PIK3CA*, and *EGFR*), genes responsible for cellular differentiation (*NOTCH1*), and Wnt signaling pathway regulator gene *FAT1*, a protocadherin modulator of adhesion and invasion^8,14,18^ (Fig. 1). From the TCGA data, the two most predominant gene alterations and mutations reported were *TP53* and *CDKN2A*, which were largely absent in HPV^+^ tumors, however, both HPV^+^ and HPV^−^ HNSCC tumors shared similar amplifications in *PIK3CA* and *SOX2* genes.^8^

The TCGA further categorized HNSCC into four gene expression subtypes: basal, mesenchymal, atypical, and classical. In accord with previous findings, the basal subtypes were associated with amplification of chromosomes 11q and 13q, Notch downregulation, and minimal alterations in the *SOX* gene. The mesenchymal group was associated with a high expression of CD56, and a low frequency of mutations in human leukocyte antigen (HLA) class I. HPV-driven tumors were prominent in the atypical subgroup, with gain in PIK3CA mutations. The classical subtype was associated with a history of heavy smoking, mutation in p53, 3q chromosome amplification, and loss of function of CDKN2A gene were prevalent.^8^ In a more recent study, HNSCC were further categorized into five groups. Non-HPV basal, HPV classical, non-HPV classical, HPV mesenchymal, and non-HPV mesenchymal. On correlating the findings, these groups were further classified into three prime subgroups. The basal subgroup, possessing a hypoxic tumor and microenvironment, with an absence of an immune response. The classic subgroup, related to heavy smoking, and an amplification in genetic mutations. Finally, the mesenchymal subgroup, with a high expression of mesenchymal transition markers and immune cells.^19^ Recent single-cell RNA-sequencing (scRNA-seq) findings from the same group have demonstrated that the mesenchymal subgroup and the basal subgroup with extensive stromal TME share many characteristics on the level of gene expression. Therefore, adoption of a new classification, malignant-basal, which would combine the two prior classifications, may more accurately reflect real biological differences between types of HNSCC tumors.^20^

### Abnormal cancer cell proliferation and cell-cycle regulators p53 and Rb

The regulation of the cell cycle is often altered in neoplastic cells to overcome growth arrest or cellular senescence and to attain unlimited replicative potential.^21^ Mutation of tumor suppressor gene, *TP53*, is one of the earliest identified and most common genetic alterations discovered in over 50% HNSCC specimens examined.^8,22^ In p53 wild-type HNSCC tumors, the p53 functional pathway may be deactivated by other mechanisms, such as an increase in expression of MDM2, a negative regulator of p53, or deletion of CDKN2A, which can block p14/ARK, a suppressor of MDM2. In HPV^+^ HNSCC cases, the expression of viral oncoproteins E6 and E7 leads to binding and targeted destruction of E6 to p53 and E7 to Rb.^6,23^ Overall, these findings suggest that in at least 80% of HNSCC cancers, the p53 pathway is downregulated or inactivated.^22^ Furthermore, inactivation of the *TP53* gene in HNSCC also accounts for the clinical behavior of tumors in response to therapy, as functional mutations in *TP53* have been reported to have a significant impact on survival rate.^21^

The vital role of Rb pathway is evidenced by the inactivation of CDKN2A, encoding the cell-cycle modulators p14/Arf/INK4B and p16/INK4A, in a large proportion of head and neck malignancies. It has previously been shown that mutations in CDKN2A gene are evident in over 75% of HNSCC specimens studied.^24,25^ In addition, Leemans et al. reported an amplified expression of up to 80% of cyclin D1 in HNSCC.^22^ These findings suggest that both inactivation of CDKN2A and overexpression of cyclin D1 may be necessary to produce aberrant cell-cycle progression through cyclin-dependent kinases 4 and 6. Furthermore, the gain in p16/INK4A expression has been shown to be an independent predictor of patient outcome in oropharyngeal carcinoma.^26^

### Notch signaling and cancer progression

The Notch signaling pathway has been associated with multiple biological functions, including modulation of regeneration and self-renewal, exit from the cell-cycle through amplification of p21/CDKN1A expression, and cell differentiation and survival.^27^ Perhaps the most novel finding to emerge from next-generation sequencing techniques investigating HNSCC tumors is the discovery of inactive mutations in the NOTCH1 gene in 12–15% of the cases, making NOTCH1 the second most frequent mutation occurring in HNSCC after p53.^14,17,28^ Mutations and translocations of NOTCH gene have been reported to have pro-tumorigenic implications in several malignancies including B-cell lymphoma, T-cell acute lymphoblastic leukemia, and chronic lymphoblastic leukemia. However, Notch signaling has also been shown to exhibit tumor-promoting roles in HNSCC development.^29,30^ The role of Notch signaling in HNSCC development and progression is controversial and needs further investigations.

### PIK3CA

The phosphoinositol-3-kinase signaling pathway gene *PIK3CA* is associated with both rapamycin (mTOR) and EGFR, and responsible for modulating cell growth, death, and proliferation. It is regularly altered in HNSCC, and commonly mutated in HPV-driven tumors.^8,31,32^ Due to the common alteration in PI3K-AKT-mTOR detected in HNSCC, several preclinical and clinical studies have attempted to target this pathway; however, the results still remain inconsistent.^31,33^ Pan-PI3K inhibitor buparlisib and alpha-specific PI3K inhibitor alpelisib have been widely investigated in conjunction with anti-EGFR drug cetuximab for treatment of HNSCC.^34–36^

PIK3CA and SOX2 gene are both expressed on the distal locus 26 of chromosome 3q and are significantly upregulated in HNSCC.^37^ There is increasing evidence that genetic alteration and oncogenic mutations manipulate the immune response in tumors. Recently, SOX2 has also been reported to play a key role in HNSCC immune escape.^38,39^ In a study conducted on immunocompetent mice, SOX2 expression in HNSCC tumor cells decreased CD8^+^ T-lymphocyte infiltration and promoted tumor growth through suppression of stimulation of interferon genes (STING)-dependent interferon-I-mediated signaling. These findings suggest that SOX2 compromised an antitumor immune response in HNSCC development.^38,39^

## TME in HNSCC

The importance of the TME in cancer progression is not a hypothesis that has only recently been proposed. In fact, it was first highlighted by Rudolph Virchow in 1863. In his study, leukocytes were identified surrounding cancer cells, and from this, he hypothesized that cancer originated from chronic inflammation. However, his study only focused on immune cells and did not examine or consider other components of the TME.^40^ When Paget proposed the “seed and soil” hypothesis in 1889, it included all the components of the TME. Moreover, Bissell et al. concluded that the role of the TME was as critical as genetic predisposition in the development and progression of malignancies.^41^ For the past years, understanding the fundamental role of TME in regulating HNSCC development has been the focus of numerous studies. Investigating the function of the main cellular components of the TME and how these cells interact with each other and with the malignant cellular counterpart via complex communication networks through the expression of growth factors, cytokines, and chemokines has been gaining more attention.^42–44^ Extensive characterization of the TME is of prime importance in establishing reliable prognostic markers and new advanced modern therapeutics for HNSCC.

The TME in HNSCC is composed of heterogeneous nonmalignant cells integrated in a complex ECM, collectively making up the tumor stroma. Constant crosstalk between cancer cells and surrounding stroma is continuously maintained, allowing tumor cell activation of the microenvironment, which results in the transmission of paracrine signals increasing neoplastic cell proliferation and metastasis. The fundamental cellular components of the TME are immune cells, fibroblasts, mesenchymal cells, hematopoietic and bone-marrow-derived cells, vascular endothelial cells, and nerves^42,43^ (Fig. 1). These non-neoplastic cells are estimated to make up as high as 90% of the tumor mass.^44^ Although non-neoplastic cells within the TME may have protective roles in restricting tumor progression, there is increasing evidence that these cells play a functional role in tumor growth and dissemination.

### Cancer-associated fibroblasts (CAFs)

The TME is largely composed of supportive stromal cells, including CAFs. CAFs share common phenotypic characteristics to myofibroblasts found in wound contraction during healing and in granulation tissue during fibrosis. CAFs are heterogeneous in nature as they differentiate from different cell types (mesenchymal stem cells, fibrocytes, pericytes, and epithelial cells through epithelial mesenchymal transition (EMT)) and can also be divided into distinct subpopulations.^45–47^ Various markers have been used to identify CAFs including alpha smooth muscle actin, vimentin, fibroblast activation protein (FAP), and fibroblast-specific protein 1.^45^ Unfortunately, due to the vast genetic diversity of fibroblasts, these markers are not specific. In HNSCC, myofibroblastic differentiation of normal oral fibroblasts is initiated through transforming growth factor β (TGFβ)-dependent pathways, which has been shown to be amplified in the presence of interleukin (IL) 1β.^47^ Furthermore, neoplastic cells secrete FAP and platelet-derived growth factor A (PDGF-A), which is suggested to contribute to the accumulation of CAF in TME.^45,46^

These nonmalignant cells have significant tumor-supporting roles in the HNSCC, through their expression of various cytokines and growth factors. CAFs have been reported to promote angiogenesis, tumor cell proliferation, and regional metastasis.^45–47^ CAFs in HNSCC appear to be strongly correlated with invasion, cancer relapse, treatment resistance, and poor patient outcomes.^48,49^ In HNSCC, CAFs have been shown to promote an immunosuppressive TME by secreting significantly amplified levels of IL-6, TGFβ1, tumor necrosis factor (TNF), vascular endothelial growth factor (VEGF), and CXCL-8, suppressing T lymphocyte via PD-1 and its ligand PD-L1 checkpoint inhibitor, further initiating T-lymphocyte apoptosis, and T regulatory lymphocyte proliferation and infiltration^50^ (Fig. 1).

### Inflammation in the TME

A state of inflammation enhances the development of malignancy.^51,52^ Inflammation in cancer has previously been reported to trigger genetic instability in cancer cells.^51^ There is a large amount of evidence indicating that the TME is rich in infiltrating immune cells of various types. Immune cells have been reported to express cytokines, growth factors, and proteolytic enzymes (matrix metallopeptidases (MMP), TNF-α, ILs, VEGF, TGFβ, cyclooxygenase 2, and CXC motif ligand 8) influencing metabolic changes altering the TME phenotype. Further supporting this data, the chemotactic signaling of VEGF, monocyte chemotactic protein 1, and macrophage colony-stimulating factor-1 resulted in the accumulation of tumor-associated macrophages (TAMs) in the TME, correlating with an unfavorable prognosis in a range of malignancies.^43,53,54^ Although the presence of inflammation may be beneficial in eliminating neoplastic growths, an immune response that is regulated by cancer cells produce an opportunity for the development of malignant cells that are capable of escaping the destructive effects of the immune system. In HNSCC, local and systemic inflammatory responses have recently been reported to be dysfunctional.^55,56^ The TME is composed of various cells from both adaptive (T and B lymphocytes) and innate (TAMs, tumor-associated neutrophils (TANs), myeloid-derived suppressor cells (MDSCs), mast cells, dendritic cells (DCs) and natural killer (NK) cells) immune systems. Numerous studies have demonstrated the important role immune cells play in neoplastic lesions. These comprise tumor suppressor immune cells, CD8^+^ cytotoxic T, T helper 1 (Th1), T helper 17 (Th17), M1 TAMs, N1 TANs, DCs, and natural NK cells, which are frequently associated with a good prognosis. Immunosuppressive cells, such as T helper 2 (Th2), M2 TAMs, MDSCs, and N2 TANs, are often correlated with an unfavorable outcome.^55–57^

In solid tumors, the distribution of immune cells in the TME is heterogenous (Fig. 1). Mast cells and TAMs are found directly surrounding tumors. Mast cells express a ray of Toll-like receptors (TLRs) and have the ability to respond to external signals secreting inflammatory cytokines including IL-6 and IL-13, which contribute to both adaptive and innate immune system activation. TAMs stimulate angiogenesis and tumor cell proliferation. M2 TAMs secrete pro-tumor factors (IL-4, IL-6, IL-10, IL-13, EGF, and VEGF), while tumor suppressor M1 TAMs express antitumor factors (IL-12, major histocompatibility complex (MHC) class II, and TNF-α) (Fig. 1). Cytotoxic NK cells downregulate MHC class I as a way to avoid immune surveillance are commonly identified within the stroma. T lymphocytes play a key role in antigen-specific immune responses. These cells regulate the functions of antigen presenting cells (APC) and are capable of directly killing targeted cancer cells. T lymphocytes are frequently evident at the periphery of the TME and in lymph nodes.^57^

### T lymphocytes

T lymphocytes can be divided into two separate groups: CD4^+^ and CD8^+^ T lymphocytes. In addition, CD4^+^ T lymphocytes are further divided into two subgroups exhibiting distinct characteristics: regulatory T (Treg) and helper T (Th) cells. Cytotoxic CD8^+^ T lymphocytes have important antitumoral activity by recognizing and destroying tumor cells. Presence of CD8^+^ T lymphocytes infiltration is therefore related to a favorable prognosis.^58^ Furthermore, Th cells help to support CD8^+^ T lymphocytes to eliminate tumor cells. In contrast, Tregs have immunosuppressive effects and can inhibit the functions of cytotoxic CD8^+^ T lymphocytes (Fig. 1). Moreover, cell count impairment in circulating and tumor-infiltrating cytotoxic CD8^+^ T lymphocytes have also been reported in patients with HNSCC.^57,59^ An amplification in circulating Tregs in patients with HNSCC has also been considered a poor prognostic factor.^60,61^ In oropharyngeal cancer, it has been shown that while increased levels of cytotoxic CD8^+^ T lymphocytes is a favorable prognostic factor, the effect of Tregs still remains controversial.^62^

### TANs

TANs constitute a significant group of tumor infiltrating immune cells that are evident in many solid tumors, including HNSCC.^63,64^ Recently the role of TANs in tumorigenesis has been studied in murine models and remains relatively poorly investigated in humans. TANs have both antitumor and pro-tumor functions depending on the stage of tumorigenesis^65,66^ (Fig. 1). TANs have been reported to stimulate neoplastic cell proliferation, TME matrix degradation, angiogenesis, promoting metastasis.^65^ In contrast, TANs also have antitumor functions, triggering CD8^+^ T-lymphocyte proliferation and initiating malignant cell apoptosis.^67,68^ Following the discovery of the diverse effects of TANs on carcinogenesis in murine models, the anti-tumorigenic N1 neutrophils and the pro-tumorigenic N2 neutrophils were proposed.^69^

Elevated levels of either circulating or TME-infiltrated neutrophils were reported to be associated with an unfavorable patient outcome.^70^ Accordingly, the neutrophil-to-lymphocyte ratio (NLR) was introduced, which measured the number of neutrophils in circulation in relation to the number of leukocytes and is considered as a prognostic factor in HNSCC, esophageal, breast, prostate, gastric, colon, and liver cancers.^59,71^ A recent study investigated NLR in patients with HPV^−^ and HPV^+^ HNSCC. Interestingly, NLR was significantly lower in HPV^+^ tumors than in HPV^−^, and a worse patient survival rate was related to an increased NLR in HPV^+^ patients.^72,73^

### MDSCs

MDSCs are heterogenous immature immunosuppressive cells and can be divided into two distinct groups: monocytic and granulocytic. The latter has been reported to resemble neutrophils; however, lacking immunosuppressive and antitumor features.^74^ MDSCs are recruited via chemotaxis to the TME by IL-1β, IL-6, IL-10, granulocyte macrophage-colony-stimulating factor (GM-CSF), and VEGF.^75^ These cells can conduct various functions in the TME, such as promotion of angiogenesis and activation of Tregs and Th2 cells. Moreover, MDSCs compete with APC such as DCs and TAMs and induce deprivation of cysteine, which is essential for T-lymphocyte activation. MDSCs also produce arginine, an essential amino acid that inhibits T-lymphocyte response. In addition, metabolism of arginine leads to the production of nitric oxide and reactive oxygen species suppressing T-lymphocyte receptor and HLA communication, further inhibiting T-cell activation.^76^ Furthermore, their expression of PD-L1 that binds to PD-1 on T lymphocytes interferes with T-cell-mediated immune mechanism, promoting a pro-tumor response^77^ (Fig. 1). Recently, these cells have been reported to suppress NK cell activity, decreasing the efficacy of NK cell-based immunotherapy in HNSCC.^78^

### TAMs

Macrophages that infiltrate in the TME of tumors are defined as TAMs. These cells originate from blood monocytes derived from bone marrow hematopoietic stem cells.^79,80^ TAMs play a major role in ECM degradation and in reorganization of the TME, promotion of tumor cell motility, and initiation of angiogenesis. TAMs are recruited to the TME by IL-10, M-CSF, PDGF, VEGF, and chemokines.^79^ TAMs exhibit a characteristic phenotype and have been shown to express tumor-promoting factors VEGF^80^ and EGF^81^ and induce angiogenesis, while subsequently stimulating invasion and metastasis.^82–84^ Macrophages can be classified according to their activity, as classically (M1) or alternatively activated (M2).^82^ In conventional immunological reactions, M1 macrophages have proinflammatory characteristics and are activated through Th1 cytokines, interferon gamma (IFN-γ), and lipopolysaccharide in response to the presence of pathogens. M1 macrophages are characterized by high expression of IL-12, MHC class II, and TNF-α (Fig. 1). Conversely, during tissue wound repair, humoral, or in a pro-tumorigenic response, M2 differentiation is induced through Th2 cytokines IL-4 and IL-13, promoting angiogenesis by expressing amplified levels of IL-10, and distinctive low levels of MHC class II and IL-12.^43,82,85–87^

TAMs are recruited into tumors with a hypoxic environment and therefore frequently detected in HNSCC. In a meta-analysis study, substantial evidence showed that a high level of TAMs in HNSCC was related to an overall poor survival rate.^88^ Macrophage polarization leading to increased expression of immunosuppressive cytokines is a critical mechanism by which tumors escape immune surveillance.^89^ Current potential anticancer therapies have therefore focused on targeting TAMs and influencing repolarisation to M1 macrophages.^79,90^

### NK cells

In recent years, evidence has supported that NK cells play a key role in initiating adaptive immunity through their prompt expression of IFN-γ, which activates and promotes infiltration of Th1 cells and MDSCs^91^ (Fig. 1). NK cells contribute to both adaptive and innate immune responses. These cells are involved in cell recognition and balancing between stimulatory and inhibitory signals through the interaction with MHC class I on the surface of targeted cancer cells. However, the absence of MHC class I expression or the amplification of NKG2D ligands, which are commonly expressed by neoplastic cells can influence tumor cells susceptibility to NK cell-mediated lysis. NK cells are capable of directly targeting cells through the secretion of perforin and granzyme B to induce programmed cell death. In addition, NK cells release cytokines (IL-2, IL-12, IL-15, IFN-γ, and TNF-α) further triggering an adaptive immune response.^91–93^ However, even if they gather particularly in the stromal TME, these immune cells are not commonly evident in direct proximity to cancer cells. The potential of NK cells in influencing adaptive immunity as well as their innate cytotoxicity toward malignant cells makes these cells a prime target for cancer immunotherapy. In HNSCC patients, the number of NK cells were reported to be significantly increased and correlated with a favorable prognosis.^92,93^ As a result, investigating NK checkpoint receptors has gained remarkable interest in cancer immunotherapy.^78,91,94^

### Cytokines modulating the TME

The roles of cytokines in cancer have been well-studied and these cytokines have been divided into three groups based on their functions: regulation of tumor cell proliferation, a marker of prognosis, and potential targets of immunotherapy.^95^ HNSCCs express TGFβ, which is a key cytokine in Treg cell activation and NK cell suppression.^96^ In addition, IL-6 and IL-10 signaling have been reported to have tumor-promoting functions. These cytokines induce signal transducer and activator of transcription 1 suppression, enhancing immune evasion in HNSCC.^97^ Inhibition of T lymphocytes, TAMs, NK cell, neutrophil activation, and DCs maturation by IL-6-mediated STAT3 activation has been correlated with poor survival and recurrence of HNSCC.^98^ Other immunosuppressive signaling pathways such as IL-10 also involve the transcription factor STAT3, resulting in a decrease in IL-12 expression,^99^ and differentiation of Treg cells^100^ in the TME. Moreover, VEGF, the key promoter of angiogenesis, has been reported to be overexpressed in the majority of HNSCC,^101^ and has been shown to supress DC maturation, which results in T-lymphocyte inactivation and dysregulation.^102^ Furthermore, TLRs stimulate the expression of proinflammatory cytokines such as TNFα and IFN-γ resulting in a Th1 lymphocyte response.

In HNSCC, an increase in peripheral Treg cells leads to a state of immunosuppression.^103^ These high levels of Treg cells were inversely proportional to CD8^+^ T lymphocytes,^104^ and were also noticed to significantly amplify following oncologic treatment and associated with anti-PD-1 immunotherapy resistance and overall outcome.^105^ Interestingly, a subpopulation of suppressor CD4^+^CD25^+^ Treg cells have been associated with an altered autoimmune system promoting tumor growth.^106^ Following binding of IL-12 to the CD25 receptor, immune suppressor cytokines TGFβ and IL-10 were produced to further stimulate tumor cell proliferation.^107^

On the other hand, TAMs in the TME that have a M1 phenotype also have antitumor effects through their expression of IL-12, TNF-α, and IFN-γ. Alternatively, TAMs can produce IL-4 and IL-13 influencing a Th2 lymphocytes response, promoting tumor growth. Unfortunately, in HNSCC patients, elevated levels of Th2 cytokines IL-4, IL-6, and IL-10 generate an immunosuppressive state, creating an obstacle for targeted immunotherapy in oncogenic treatments.^107^ Increasing expression of EGF, IL-6, and IL-10 is associated with unfavorable patient outcomes.^108^

### Hypoxia

Throughout tumorigenesis, and as a result of an increase in malignant cell proliferation and a deficiency in vascular supply, regions of hypoxia develop. While hypoxia is often considered to have antitumor effects, a number of studies have shown otherwise.^109,110^ Hypoxia-inducible family (HIF) expression in hypoxic regions, which can be activated by EGFR signaling, have been reported to regulate cellular functions such as metabolism, proliferation, and remodeling.^111^ A significant increase in EGFR expression is commonly observed in HNSCC, particularly HPV^−^ patients, and is correlated with a poor outcome.^112^ Interestingly, not only has hypoxia been reported to induce EGFR expression, it has also been shown to promote immunosuppression, through the expression of cytokines, impeding T-lymphocyte proliferation, recruitment of immunosuppressive cells, and upregulation of PD-L1.^109,113^

## Mechanisms of HNSCC cells escaping immune surveillance

### Cancer immunosurveillance and immunoediting

The concept that the immune system plays an important role in malignancies was first proposed by Ehrlich in 1908. Burnet further introduced the hypothesis of cancer immunosurveillance, proposing that cancer cells express antigens that are distinct from normal cells, and accordingly, are potential targets for immune clearance.^114^ This hypothesis was abandoned for decades due to conflicting scientific data. However, in 1975, Kiessling et al. were the first to discover NK cells, which appeared to confer innate immunity against cancer cells.^115^ Furthermore, immunodeficient patients had an increased risk of developing HPV-related HNSCC.^116,117^ In addition, organ transplant recipients under immunosuppressive medications had a higher risk of tumor development, including HNSCC, with unknown viral etiology.^118^

Typically, cancer cells can be recognized as abnormal and be targeted by immune cells. Cancer immunoediting is a process utilized by tumor cells to escape immune surveillance. Malignancy development occurs after neoplastic cells gain a means to evade the immune system.^119^ Cancer cells have generated various mechanisms to escape immune surveillance such as loss of MHC and expression of immunosuppressive factors such as IL-6, IL-10, TGFβ, prostaglandins, and Fas ligand, leading to apoptosis of tumor-infiltrating lymphocytes.^120,121^ In addition, neoplastic cells are capable of recruiting TAMs by secreting chemokines, colony-stimulating factor (CSF-1), and VEGF.^83,122^

Numerous studies have reported a negative correlation between patient outcome and level of TAMs infiltrating into the TME in HNSCC.^82–86^ These findings are highly dependent on TAM expression of PDGF, TGFβ, EGF, IL-1, IL-6, and TNF-α, which generates a favorable environment for tumor growth. Hypoxia has been reported to manipulate the behavior and functionality of immune cells in many cancers including HNSCC, and a salient feature of tumorigenesis is evasion of immune recognition and under hypoxic conditions.^123–125^ Hypoxic conditions in cancer have been known to generate various immune suppressive components, such as IL-10 and TGFβ. This process could induce the differentiation of TAMs into M2 macrophages, diminishing antitumor effects.^124,125^ Under hypoxic stress, TAMs secrete TNF-α, IL-1, IL-6, IL-8, VEGF, GM-CSF, and TGFβ, promoting angiogenesis, and MMP, promoting cancer cell invasion. In addition, HIF-1α has been shown to regulate cytokine expression and survival of CD4^+^ and CD8^+^ T lymphocytes by generating increased levels of IFN-γ thereby prompting an antitumor response.^126^ Another main role of HIF-1α is the evasion of NK cell-dependent tumor cytolysis. Stress-regulated major histocompatibility class I chain-related protein A and B (MICA/B) and heat shock protein 70 (Hsp70) are both ligands for activated NK cells, expressed on the cell surface of tumor cells and contribute to the escape from NK cell cytotoxicity.^127^

### Characteristics of immune escape and immunosuppression in HNSCC

It has been proposed that HNSCC could escape immunosurveillance by various mechanisms. To develop effective immunotherapies, it is important to understand the different pathways in which tumor cells evade the immune response. Primarily, tumor cells reduce their innate immunogenicity, and actively suppress several cell signals such as HLA-peptide antigen interactions, cell-to-cell co-stimulatory/inhibitory signals, cytokine secretion, or expression of chemokine signals to recruit immune cells to the TME regulating the antitumor immune response.

The MHC is a key element for the immune cells to recognize abnormal cells expressing tumor antigenic peptides to T lymphocytes.^102^ Cancer cells may evade CD8^+^ T-cell recognition through downregulation of MHC, allowing unrecognition of antigen.^128^ In this process, antigens are degraded through the cytoplasmic immuno-proteasome. These antigenic peptides are transferred by antigen processing (TAP1/2) heterodimer of the antigen processing machinery (APM) to the endoplasmic reticulum, where they are linked to HLA class I heavy chains.^129^ Subsequently, cancer cells are vulnerable to recognition by NK cells. However, tumor cells are capable of developing further adaptations to avoid NK cell recognition as a result of mutations in MHC class 1 and APM.

Immune checkpoint receptors are another important factor that controls the immune system, limiting autoimmunity, and regulating inflammatory responses, which can be utilized in the TME. These receptors attenuate the effect of CD8^+^ cytotoxic T lymphocytes, which play a key role in identifying and targeting malignant cells. To trigger CD8^+^ T-lymphocyte activation, two pathways have been defined. Primarily, T lymphocytes receptor recognition and binding to MHC presented by an APC. The second pathway involves binding of B7 ligand on the APC with its receptor, CD28, on T lymphocytes, resulting in T-lymphocyte activation and proliferation. However, the regulatory counterpart CTLA-4 in T lymphocytes functions by suppressing T-lymphocyte activation, following co-stimulatory recognition of tumor antigen presented by APCs. Following recognition, TCR triggers the expression of PD-1 receptor, and secretion of IFN-γ, suppressing PD-L1 expression, switching off the tumor suppressor T-lymphocyte response.^130–132^

**Fig. 2:**
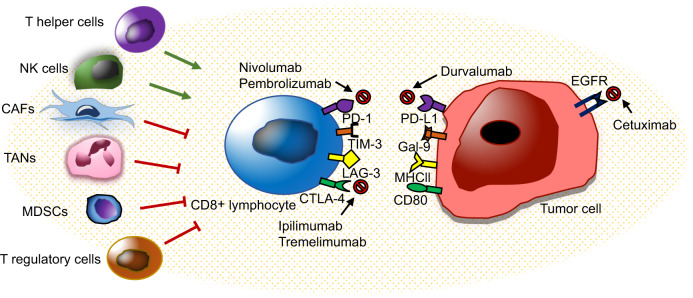
Immunoregulators and immunotherapeutic strategies in HNSCC. The inflammatory state of the TME can modulate CD8^+^ T-lymphocyte response to cancer cells. Nonmalignant cells (CAFs, TANs, MDSCs, and Tregs) in the TME have been reported to inhibit CD8^+^ T-lymphocyte, while NK cells and Th lymphocytes both have CD8^+^ T-lymphocyte supportive functions. HNSCC is infiltrated with immune cells. Cetuximab, an EGFR inhibitor, was the first immune targeted therapy to be approved in HNSCC. PD-1 immune checkpoint inhibitor, nivolumab, and pembrolizumab have recently been granted approval for use in advanced or metastatic HNSCC. Immune checkpoint inhibitors (PD-1, PD-L1, CTLA-4, TIM-3, and LAG-3) are important therapeutic targets for treatment and prevention of HNSCC

Various receptors, including lymphocyte-activation gene 3 (LAG-3), CTLA-4, T-cell immunoglobulin mucin protein-3 (TIM-3), and PD-1 have been recognized to be expressed on the surface of T lymphocytes (Fig. 2). In HNSCC cells, PD-1 and PD-L1 ligand have been shown to be amplified, leading to a cytotoxic T-lymphocyte dysfunction.^133^ LAG-3, expressed on NK cells, CD4+ and CD8+ T lymphocytes, B lymphocytes, and DCs, which have been reported to stimulate Treg cell activation.^134,135^ TIM-3 is expressed on NK cells and T lymphocytes, and when co expressed with PD-1, results in exhaustion of CD8^+^ T lymphocytes.^136^ In addition, an antitumor CD4^+^ and CD8^+^ T-lymphocyte immune response was reported following TIM-3 blockade in an HNSCC murine model.^137^ To develop effective immunotherapies, understanding the precise mechanisms in which tumor cells escape immune surveillance is critical.

Cancer stem cells (CSCs), also known as tumor-initiating cells, play a critical role in the initiation, growth, metastasis, and therapy resistance of HNSCC. Since CSCs, also known as tumor-initiating cells, are considered as the origin of cancerous tissues, CSCs may develop intrinsic mechanisms to evade immune surveillance, which is poorly understood. Very recently, we identified that CSCs utilized the immune checkpoint molecule CD276 (B7-H3), but PD-1 (also known as CD274 or B7-H1), to evade immune surveillance in a 4-nitroquinoline 1-oxide-induced mouse HNSCC. Interestingly, CD276 was uniquely expressed in the invasive front and periphery tumor nest of HNSCC and served as a shield against lymphocyte infiltrations. Anti-CD276 antibodies significantly inhibited HNSCC invasive growth and lymph node metastasis. Moreover, we found that HNSCC patients with high CD276 expression had a poor prognosis and CD276 expression was reversely associated with lymphocyte infiltrations in human HNSCC tissues.^138^

### Immunotherapeutic implications in HNSCC

HNSCC is one of the most frequently immune-infiltrated cancer types with Treg, and NK cells.^93,102^ A recent review has discussed the efficacy of immune checkpoint inhibition in these tumors.^93^ Within the past few decades, the only targeted therapy that has been approved for HNSCC therapy is the EGFR inhibitor, cetuximab. Recently, monoclonal antibodies (MoAb) nivolumab and pembrolizumab against PD-1 and atezolizumab against its ligand PD-L1, were granted approval for use in combination treatment with conventional oncotherapy for advanced or metastatic HNSCC^16^ (Fig. 2). The heterogeneous phenotypes present within HNSCC demonstrate diverse genetic aberrations over a complex mutational landscape, making their response to targeted therapies limited and creating an urgent demand for effective immunotherapies, which exploit the weaknesses of tumors with high mutational burden.^6,14^

Recently, MoAb treatments have become widely adopted for treating cancer. In HNSCC, several MoAb are currently under investigation. These MoAb can be divided into four groups in accordance with their mechanism of action: (1) cytokine-targeted MoAb: neutralizing antibodies bevacizumab targets VEGF and neutralizing antibodies ficlatuzumab targets hepatocyte growth factor. (2) Tumor antigen targeted MoAb: cetuximab and panitumumab antagonize EGFR, AV-203 antagonize HER3, and cixutumumab antagonize IGFR. (3) The TNF receptor family targeted MoAb: urelumab and PF-05082566 agonize CD137, and medi0562 agonize OX40. (4) Immune checkpoint-targeted MoAb: nivolumab, pembrolizumab targets PD-1, durvalumab targets PD-L1, and ipilimumab and tremelimumab targets CTLA-4 (Fig. 2). Numerous studies have shown amplified EGFR expression in HNSCC, and for this reason, cetuximab is still the most frequently studied drug. Cetuximab might promote tumor cell destruction by cytotoxic phagocytosis or cytolysis of tumor cells.^139,140^ In addition, emerging evidence has supported the combination of cetuximab with immune checkpoint inhibitors in HNSCC therapy. Cetuximab therapy has been shown to alter the expression of tumor infiltrating lymphocytes, particularly increasing the recruitment of PD-1 and TIM-3 positive CD8^+^ T lymphocytes.^137^

Despite the recent progress in immunotherapy, the responsiveness and overall survival rate to immunotherapy is not ideal and still remains to be improved.^141–143^ The TME of HNSCC comprises diverse cells, receptors, signaling pathways, which should be targeted simultaneously through combination therapy. There is increasing evidence suggesting that CSCs express diverse cytokines and growth factors that stimulate an immunosuppressive microenvironment in cancer,^144–146^ and are capable of escaping T-lymphocyte attack, while also being resistant to immunotherapy.^147^ In HNSCC, CSCs have been reported to express amplified levels of PD-L1.^148^ However, despite the recent advancements in cancer immunotherapy, there is a lack of studies investigating the effect of immune checkpoint MoAb on CSCs. Targeting CSCs has been recommended in the development of novel next-generation anticancer drugs.^147,149^

In our recent work, we have shown that BMI1^+^ CSCs play a critical role in HNSCC tumor cell initiation, proliferation, and cervical lymph node metastasis in murine models. Upon targeting these CSCs, the tumors were significantly responsive to chemotherapy, and cervical lymph node metastasis was obliterated.^150^ Moreover, there is growing evidence that these stem cells may manipulate the host immune response in cancer, promoting an immunosuppressive TME.^146,151^ We have further investigated BMI1^+^ CSC responses following a combination of cisplatin and PD-1 immunotherapy. Unexpectedly, we found that BMI1 inhibition promoted the expression of CD8^+^ T-cell-recruiting chemokines by inducing DNA damage and erasing repressive H2A ubiquitination. The combination of anti-PD-1 immunotherapy and BMI1 inhibition significantly increased CD8^+^ T-lymphocyte infiltration in the TME, thereby inhibiting HNSCC invasive growth and metastasis.^145^ These novel findings provide a foundation in developing combination therapies targeting CSCs and improving the immune response, which holds promise for treating diverse malignancies. Previously, we have identified that the histone demethylase KDM4A plays a critical role in HNSCC invasive growth and metastasis. Interestingly, we found that targeting KDM4A induced DNA replication stress and promoted antitumor immunity in HNSCC by activated tumor cell-intrinsic cGAS-STING signaling. Moreover, KDM4A inhibition cooperated with PD-1 blockade to suppress HNSCC growth and metastasis by recruiting CD8^+^ T cells into the TME.^152^

With the continuous advancement in innovative cancer treatments, novel approaches have recently developed involving vaccinations, oncolytic viral and adoptive cell transfer (ACT) therapy, or adoptive immunotherapy, which all show promise in HNSCC treatment. As the rising cases of HNSCC have been reported to be HPV^+^, not only did the FDA approve routine vaccination in children (ages 11–12 years), but also included males (13–21 years), and females (13–26 years) with no prior vaccination. HPV vaccination has been reported to be significantly effective in preventing viral dissemination and HPV^+^ HNSCC development.^153^ Oncolytic therapy involves utilizing viruses to target tumor cells and multiply, resulting in neoplastic cell death. In HNSCC, Talimogene laherparepvec, a biopharmaceutical drug bioengineered from the virus strain herpes simplex type 1 to secrete GM-CSF, encouraging a tumor suppressor immune response has been investigated. Data from preliminary clinical trials have reported a significant response following treatment.^154,155^ ACT therapy involves isolating T cells from patients, culturing and expanding them ex vivo, and then administering them back into the patient. These strategies generate a long-term antitumor immune response, and have been granted FDA approval for hematologic malignancies,^156^ but are still undergoing phase I trials in HNSCC.^157–159^

### HNSCC heterogeneity and resistance to immunotherapy

In spite the fact that HNSCC arise from a homogenous squamous mucosal lining of the oral cavity and upper aerodigestive tract, however, these tumors have been defined to be extremely heterogenous.^57^ Advanced research comprising genetic, epigenetic analyses and evidence of cellular heterogeneity in the TME, in particular the immune landscape, has added to the complexity of tumorigenesis. Tumor progression further leads to various genomic alterations. This mechanism provides tumor cells with superior proliferation and survival capabilities, in addition to alterations in genes responsible for evading immune recognition and T-lymphocyte obliteration. Very recently, we showed that that CD276 blockade changed SCC heterogeneity with decrease of partial EMT by scRNA-seq. In vivo lineage tracing revealed that anti-CD276 effectively eliminated CSCs in a CD8^+^ T-cell-dependent manner.^138^ In the future, it will be interesting to examine how immune check blockade modifies the landscape of HNSCC heterogeneity.

Although immune checkpoint blockage has been approved by the FDA for the treatment of metastatic HNSCC, however, most patients do not exhibit high response rates.^141–143^ On account for the heterogeneity in HNSCC, challenges still remain in identifying patients that will respond to immunotherapy. Tumors have the ability to evade both innate and adaptive immunosurveillance, thereby becoming ineffective to immunotherapy. Immune checkpoint blockade is highly dependent on the adaptive arm of immunity or the ability of cytotoxic T lymphocytes to recognize and target tumor cells. Tumor cells that are weakly antigenic are highly likely to evade immunosurveillance leading to tumor progression. An explanation for the low success rates of these therapies is due to the development of resistance. Terminal exhaustion of tumor antigen-specific T lymphocytes have been reported, making immune checkpoint blockage ineffective. Moreover, T-cell exhaustion can also be induced by TAM (M2), Treg, and MDCSs through the expression of immunosuppressive cytokines promoting an immune suppressive TME.^55^ Furthermore, impairment in T lymphocytes durability/memory may lead to acquired resistance and tumor relapse following immune checkpoint blockade therapy termination.^160^ Understanding how the genetic profiles and immune phenotypes in HNSCC develop over time may prove beneficial.

Mechanisms of innate and adaptive resistance to immunotherapies are not fully understood. Innate and adaptive arms of the immune system are in constant evolution during tumor development, progression, and following conventional cancer treatments (chemotherapy and radiation) or immune therapy.^160^ Targeting innate immune cells independently has shown promising results in both preclinical and clinical trials.^79,90,92,94^ Recently developed NK cell therapies have demonstrated significant tumor suppressive capabilities in preclinical studies.^78,94^ NK cells can be activated in the absence of MHC to recognize and target malignant cells. In these cases, a promising approach would be NK cell-based monotherapy or in combination with T-cell-based immunotherapy. Moreover, the concept of trained innate immunity (TII) or innate immune memory has been gaining more interest. Recent studies have demonstrated a shift in the TME, in particular TAN, to a more antitumor phenotype after TII therapy. Significant reduction in tumor growth and a long-term functional reprogramming of TAN to exhibit tumor suppressive roles was reported following single administration.^161^ To overcome these obstacles, meticulous understanding of HNSCC heterogeneity will help in the development of new immunotherapeutic applications to improve patient outcomes. Therefore, further investigation of innate immune cells is of prime importance in developing innovative cancer therapeutics.

## Conclusion

HNSCC immunology is a complex and rapidly developing area. The main focus for previous research was to identify genetic mutations and aberration in tumor cells. However, understanding the crosstalk between cancer cells and the host immune system is crucial in effectively treating HNSCC. Tumor cells adapt several mechanisms to escape immune surveillance, promote tumor cell proliferation, survival, and metastasis. Defining cellular and molecular signaling pathways associated with resistance to immune checkpoint blockade may allow the development of novel immunotherapies to overcome these resistance mechanisms. The standard of care for HNSCC is currently evolving. A decade after cetuximab approval, anti-PD-1/PD-L1 checkpoint inhibitors are the first drugs to demonstrate prognostic benefits for the treatment of platinum-refractory recurrent or metastatic HNSCC and remain to be granted approval for treatment of primary and locally advanced HNSCC. Unfortunately, the response rate to immunotherapeutic still remains significantly low. Identification of predictive biomarkers in cancer therapy with immune checkpoint inhibitors is critical. Developing therapies to target a combination of markers may be necessary to benefit from immunotherapy. Therefore, ongoing trials combining immune checkpoint inhibitors with radiotherapy, cytotoxic agents, or other immunotherapies are in progress to improve response rate, duration, and opportunities in developing a potential cure.
